# Tumor cell GPX4 dictates triacylglycerol metabolism for ferroptosis susceptibility and immune evasion of NSCLC

**DOI:** 10.1093/procel/pwag009

**Published:** 2026-03-13

**Authors:** Yaxu Li, Ping Wang

**Affiliations:** Tongji University Cancer Center, Shanghai Tenth People’s Hospital, Shanghai Key Laboratory of Signaling and Disease Research, School of Life Sciences and Technology, Tongji University, Shanghai 200092, China; Tongji University Cancer Center, Shanghai Tenth People’s Hospital, Shanghai Key Laboratory of Signaling and Disease Research, School of Life Sciences and Technology, Tongji University, Shanghai 200092, China

**Figure 1.**
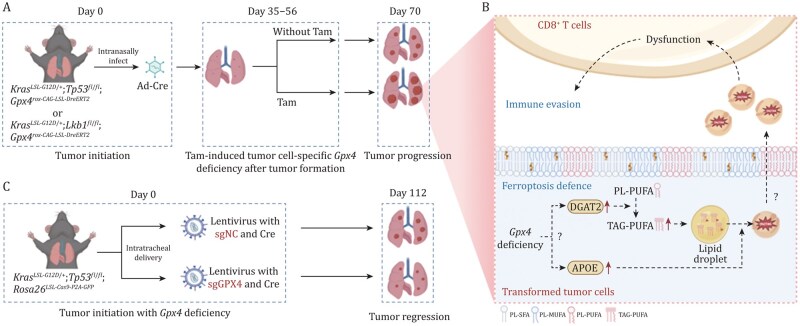
**The unexpected role of GPX4 in NSCLC mouse model**. **(**A and B). In autochthonous non-small cell lung cancer (NSCLC) models, inducible knockout of *Gpx4* in transformed tumor cells does not result in ferroptosis but instead orchestrates a profound triacylglycerol (TAG) metabolic rewiring that promotes cancer progression by evading ferroptosis and suppressing CD8^+^ T cell immunity. (C) Knockout of *Gpx4* in the beginning of tumor initiation of NSCLC mouse model results in ferroptosis and tumor regression.

The ferroptosis regulator glutathione peroxidase 4 (GPX4) has long been viewed as a guardian against lipid peroxidation, with its inhibition heralded as a promising therapeutic strategy to trigger iron-dependent cancer cell death (Yang et al., 2014). This paradigm, largely built on studies in cultured cells and subcutaneous graft models, posits that GPX4 loss uniformly induces ferroptosis and suppresses tumor growth (Yang et al., 2014; Zou et al., 2020). However, in a recent study published, *Protein & Cell*, Wang et al. (2025) demonstrate that in autochthonous non-small cell lung cancer (NSCLC) models, inducible knockout of *Gpx4* in transformed tumor cells does not result in ferroptosis but instead orchestrates a profound triacylglycerol (TAG) metabolic rewiring that promotes cancer progression by evading ferroptosis and suppressing CD8^+^ T cell immunity (Fig. 1A and 1B).

Using a dual-recombinase system (Cre-loxP and Dre^ERT2^-rox), the authors achieve a selective and temporal deletion of *Gpx4* in transformed autochthonous NSCLC cells of the *Kras^LSL-G12D/+^Lkb1^fl/fl^* (hereafter KL) and *Kras^LSL-G12D/+^T*p53^fl/fl^ (hereafter KP) mouse models. Surprisingly, such a deletion of GPX4 does not lead to ferroptosis of tumor cells or accumulation of oxidized phospholipids (oxPLs). In contrast, a recent important study shows that sgRNA-mediated knockout of GPX4 in the *Kras^LSL-G12D/+^Tp53^fl/fl^ Rosa26^LSL-Cas9/LSL-Cas9^* mouse model at the tumor initiation stage significantly induces cell ferroptosis and inhibits the progression of NSCLC (Fig. 1C) (Wu et al., 2026). It should be noted that *KRas^G12D^*-driven malignant transformation requires 2–3 weeks, while *Gpx4* deficiency causes ferroptosis within a few days (Friedmann Angeli et al., 2014; Jackson et al., 2001; Johnson et al., 2001). Therefore, the *Gpx4*-deficient lung epithelial cells may die before they undergo malignant transformation, and such a premature death might be responsible for the reduced tumor burden in the model. Certainly, this hypothesis currently lacks any experimental evidence, but it is certainly warranted to delve deeper into the reasons underlying this intriguing phenomenon.

Interestingly, knockout of *Gpx4* in the transformed tumor cells in the autochthonous NSCLC models results in the upregulation of key enzymes (such as DGAT2 and GPD1L) involved in TAG synthesis, leading to increased synthesis of TAG and oxidized TAG (oxTAG) accumulated in lipid droplets (Fig. 1B). Inhibition of DGAT1/2 attenuates (ox)TAG accumulation, promotes oxPLs generation, sensitizes tumor cells to ferroptosis, and inhibits NSCLC progression in the *Gpx4*-deficient KL tumors, which aligns with a previous study showing that lipid droplets protect the *Drosophila* glial cell niche and neural stem cells from harmful polyunsaturated fatty acid oxidation-induced ferroptosis (Bailey et al., 2015). These observations together suggest that DGAT1/2 promotes the (ox)PL-to-(ox)TAG metabolic shift to counteract ferroptotic cell death, representing another layer for ferroptotic antagonism. Furthermore, the application of oxi-lipidomics was critical in enabling the authors to distinguish between (ox)PL and (ox)TAG, especially given that the C11 probe appears to exhibit reactivity toward both.

It is enigmatic that the upregulation of *Dgat2* in syngeneic subcutaneous *Gpx4*-deficient tumors or in *in vitro* cultured *Gpx4*-deficient tumor cells is not observed, as the tumor cells and the mice in syngeneic and autochthonous models share the same background. It should be noted that the microenvironment between the syngeneic and autochthonous models is different, and that lung tumor cells lose the lung identity quickly after *in vitro* culture or subcutaneous transplantation (Quintanal-Villalonga, 2024; Snyder et al., 2013), which might be responsible for this phenomenon and requires further investigation in the future.

Importantly, *Gpx4*-deficient tumor cells also upregulated apolipoprotein E that actively promotes the secretion of TAG and oxTAG in the tumor microenvironment of the autochthonous NSCLC models (Fig. 1B). The supernatants, but not the delipidated supernatants, from *Gpx4*-deficient tumor cells potently suppress the activation and promote exhaustion of P14 CD8^+^ T cells in *in vitro* cultures. Consistent with these observations, CD8^+^ T cells exhibit reduced expression of IFNγ and granzyme B and elevated surface expression of exhaustion markers such as PD-1 and Tim-3 in the *Gpx4*-deficient KL and KP tumors compared to the *Gpx4*-sufficient counterparts. It has been shown that oxLDL (which mainly contains TAG and cholesterol) induces dysfunction of CD8^+^ T cells in a CD36-dependent manner (Ma et al., 2021; Xu et al., 2021). Whether such a mechanism applies to the dysfunction of CD8^+^ T cells in these autochthonous *Gpx4*-deficient KL and KP models has not been investigated in this study. Intriguingly, another recent study identified that extracellular GPX4 can act as a tumor immunosuppressive factor to inhibit the function of CD8^+^ T cells during ferroptosis (Liu et al., 2026). Together, these findings highlight a novel and complex role for GPX4 at the intersection of ferroptosis and tumor immunity, which warrants further in-depth investigation.

Notably, the inducible expression of *Gpx4* in transformed tumor cells downregulates the expression of *Dgat1/2*, inhibits (ox)TAG synthesis and lipid droplets accumulation, rejuvenates the activation of CD8^+^ T cells, and substantially suppresses the progression of NSCLC in the KL model. In addition, treatment with DGAT1/2 inhibitors also inhibits NSCLC progression in *Gpx4*-sufficient KL tumors. These data highlight the primary role of GPX4 in triacylglycerol metabolism through DGAT1/2 in transformed autochthonous NSCLC tumor cells *in vivo*. It should be noted, however, this study does not elucidate the detailed molecular mechanism by which alterations in GPX4 regulate TAG metabolism. Future studies are required to determine whether the enzymatic activity of GPX4 is involved and how GPX4 couples the DGAT1/2-TAG axis in this process. For instance, whether GPX4 deficiency regulates the transcription of DGAT1/2—key enzymes in TAG metabolic homeostasis—via intracellular oxidative stress-mediated responses, such as NRF2 and ATF4, remains to be elucidated. Furthermore, exploring the clinical relevance of this pathway—by correlating GPX4 expression levels with TAG metabolism and T cell exhaustion signatures in human NSCLC biopsies—will be crucial for translating these preclinical findings into novel combination therapies that simultaneously target tumor metabolism and rejuvenate antitumor immunity.
